# La significancia estadística y la relevancia clínica en la odontología

**DOI:** 10.21142/2523-2754-1002-2022-103

**Published:** 2022-06-27

**Authors:** Luis Ernesto Arriola Guillén

**Affiliations:** 1 División de Ortodoncia, Carrera de Odontología de la Universidad Científica del Sur. Lima, Perú. larriola@cientifica.edu.pe Universidad Científica del Sur División de Ortodoncia Carrera de Odontología Universidad Científica del Sur Lima Peru larriola@cientifica.edu.pe

Los odontólogos se enfrentan a decisiones clínicas muy importantes en su práctica dental diaria, por lo que permanentemente requieren de sustento científico que las respalde, claramente en beneficio de los pacientes ^1^. Sin embargo, algunas veces, en la literatura científica se encuentran resultados con diferencias estadísticamente significativas, lo que ocasiona que los clínicos deban optar por alguna alternativa de tratamiento en particular; pero resulta que podría tratarse de una diferencia significativa que clínicamente no sea relevante, y esto hace que surja en el profesional la interrogante de qué decisión tomar ^2^^,^^3^. En este sentido, el dentista debe analizar la posibilidad de que, en ciertos casos, esa diferencia significativa sea consecuencia de una dispersión en los resultados muy pequeña, con desviaciones estándar reducidas en cada grupo y, por lo tanto, los valores, al no superponerse, se hacen diferentes estadísticamente, aun cuando esto permita observar claramente promedios muy parecidos en ambos grupos. Es aquí donde el concepto de la relevancia clínica adquiere valor y el clínico puede tomar la postura de que, aunque haya resultados significativos, clínicamente estos sean considerados poco relevantes y lo lleven a tomar una decisión terapéutica final.

Sin embargo, este análisis debe ser muy concienzudo por parte de los profesionales, ya que podría generar una malinterpretación de los resultados de los artículos publicados, y obedece necesariamente a tener conocimientos claros de bioestadística y también a una experiencia clínica del tema, atributos que permitirán que la decisión sea más cercana a la correcta ^4^^,^^5^. En este sentido, los odontólogos deben capacitarse cada vez más en el área de la bioestadística, para que tengan esta herramienta como aliada en el momento de tomar las decisiones clínicas.

## References

[1] Ismail AI, Bader JD, ADA Council on Scientific Affairs and Division of Science; Journal of the American Dental Association (2004). Evidence-based dentistry in clinical practice. J Am Dent Assoc.

[2] Pandis N (2015). Statistical inference with confidence intervals. Am J Orthod Dentofacial Orthop.

[3] Pozos-Guillén A, Ruiz-Rodríguez S, Garrocho-Rangel A (2017). Fundamentals in Biostatistics for investigation in pediatric dentistry Part II -Biostatistical methods. J Clin Pediatr Dent.

[4] Pandis N (2020). Does more or new data mean that a nonsignificant result will become significant. Am J Orthod Dentofacial Orthop.

[5] Pandis N (2015). Calculating the P value and carrying out a statistical test. Am J Orthod Dentofacial Orthop.

